# Sometimes Less Is More: A Cross-Sectional Analysis of Commercially-Negotiated Price Variation for Thyroidectomy

**DOI:** 10.1097/AS9.0000000000000564

**Published:** 2025-03-12

**Authors:** Catherine B. Jensen, Mitchell Mead, Hunter J. Underwood, Andrew Ibrahim, Susan C. Pitt

**Affiliations:** From the * Department of Surgery, University of Wisconsin, Madison, WI; † Department of Surgery, University of Michigan, Ann Arbor, MI; ‡ National Clinician Scholars Program, University of Michigan, Ann Arbor, MI; § Taubman College of Architecture and Urban Planning, University of Michigan, Ann Arbor, MI.

**Keywords:** paradoxical pricing, price transparency, reimbursement, thyroidectomy, variation

## Abstract

**Introduction:**

The Hospital Price Transparency Rule requires hospitals to publicly report prices for healthcare services to enhance transparency. Among the most common thyroidectomy procedures are thyroid lobectomy (TL) and total thyroidectomy alone (TT) or with central neck dissection (TT+CND). This study aimed to examine factors associated with variations in commercially-negotiated prices for thyroidectomy.

**Methods:**

This cross-sectional analysis examined commercial price data obtained from Turquoise Health and linked to the American Hospital Association Annual Survey. Thyroidectomy procedures were categorized using Current Procedural Terminology codes (60220 TL, 60240 TT, and 60252 TT+CND, listed in increasing extent of surgery). The main outcome included intrahospital variation in commercially-negotiated prices and hospital-level factors associated with price differences.

**Results:**

Overall, 1299 hospitals (30.4%) reported commercial prices for TL and TT. In increasing order of surgical complexity, the median price (interquartile range) was $6483 ($2217–$11,443) for TL, $6732 ($2566–$11,321) for TT, and $6232 ($3118–$10,916) for TT+CND. Only 28% (n = 303) reported median negotiated prices concordant with increasing extent of thyroidectomy. Risk-adjusted mean negotiated prices found that not-for-profit hospitals had significantly lower adjusted mean prices compared with for-profit ($8266 vs $10,625, *P* = 0.022). Procedure type significantly impacted adjusted mean prices, with TT+CND having lower prices compared with TT ($8295 vs $9446, *P* = 0.001).

**Conclusions:**

The complexity of thyroidectomy is not reflected in the price-negotiated rates paid by insurers to hospitals. Most hospitals are paid less when taking on more complex procedures. These findings underscore concerns about fair reimbursement to hospitals and the potential of the Price Transparency Rule to illuminate unwarranted differences in negotiated rates.

## INTRODUCTION

Thyroid nodules are found in up to two-thirds of adults, approximately 5% to 10% of which are diagnosed as cancer.^1,2^ The management of most thyroid cancers and high-risk nodules includes surgical intervention with thyroidectomy. The recommended extent of thyroidectomy depends on multiple patient- and disease-related factors leading to either less extensive surgery with a thyroid lobectomy or more extensive total thyroidectomy with or without lymph node dissection.^3,4^ These procedures are common and performed by otolaryngologists, general surgeons, and specialty-trained endocrine surgeons.

Thyroid cancer, in particular, is a leading cause of financial toxicity with patients declaring bankruptcy at a higher rate than most studied cancers.^5^ The etiology of this financial toxicity is not well understood but is certainly multifactorial. Rising costs of healthcare and younger age at the time of diagnosis are thought to contribute.^6^ This financial toxicity has been associated with high levels of both material and psychological distress and is associated with decreased quality of life as well as increased anxiety and depression.^7,8^ In January 2021, the Centers for Medicare and Medicaid Services (CMS) enacted The Hospital Price Transparency Rule, which requires hospitals to publicly disclose prices for a wide range of healthcare services.^9^ The goal of this rule was to enhance transparency, empower patients to make informed cost decisions, and theoretically facilitate price shopping for a variety of medical services and procedures.^10^

Variations in reporting commercially-negotiated prices for thyroidectomy and factors contributing to this variation are unknown. It is important to understand the factors contributing to variation in negotiated prices due to the significant financial burden associated with thyroid cancer treatment. Therefore, we sought to examine intrahospital price variation across 3 of the most common thyroidectomy procedures—thyroid lobectomy and total thyroidectomy with or without a central neck dissection (CND). We hypothesized that significant price variation exists within hospitals by procedure and that median negotiated prices increase with the extent of thyroidectomy performed.

## METHODS

### Data Sources

To investigate price variation across thyroidectomy procedures, we utilized 2 national datasets. First, we used the 2021 American Hospital Association (AHA) Annual Survey to identify nonfederally managed hospitals (eg, Veterans Affairs) that are required to report prices under the rule. This file is one of the largest and most comprehensive national datasets documenting hospitals within the United States and is regularly used in health services research.^11,12^ The AHA Annual Survey provides hospital-level data such as hospital location, hospital type, ownership, bed size, and health system membership.

Second, we collected prices reported under the Hospital Price Transparency Rule from Turquoise Health. Turquoise Health compiles publicly reported price data from each hospital’s machine-readable files reported in accordance with the rule. Pricing data include information on the price of the service, the shoppable service code (Current Procedural Terminology [CPT]), the payer type (eg, chargemaster, commercial insurer, self-pay), and the hospital reporting the price. This dataset is updated daily as hospitals report and update prices reported under the rule. Price data utilized in this investigation were extracted from Turquoise Health on July 23, 2024. We linked pricing data to hospitals through their CMS Identification Number. This study was considered exempt by the University of Michigan Institutional Review Board.

### Hospital Price Transparency Data

To be compliant under the Hospital Price Transparency Rule, there are several price reporting requirements hospitals must meet. First, the prices reported for healthcare services must reflect care provided within the hospital and be inclusive of all “supplies, procedures, room, and board, use of the facility and other items services of employed physicians and non-physician practitioners and any other items or services for which a hospital has established a charge”.^13^ Therefore, this study reports negotiated facility fees for inpatient and outpatient procedures as well as physician fees or professional charges. Second, for each service provided by a hospital, the hospital must report a chargemaster price. In addition, CMS requires additional data to be reported for 70 services specified by CMS and 230 services for which the hospital may choose which services to report prices: gross charges (list price), discounted cash prices (self-pay), third-party price-negotiated prices (eg, commercial insurers and self-insured plans), and deidentified minimum- and maximum-negotiated charges. Because a hospital may negotiate prices with a variety of insurers for a variety of plans, several price-negotiated prices may be reported for a single service.

This investigation focused on evaluating negotiated prices with commercial insurers for thyroidectomy procedures for several reasons. First, most patients undergoing thyroidectomy procedures are commercially insured and are most likely to pay the price of a price-negotiated plan.^14^ Second, in contrast to federally-insured plans (eg, Medicare) that have predetermined reimbursements for services, commercially-negotiated prices for healthcare services may be impacted by a variety of factors, making them more subject to variation. Finally, patients with commercial insurance may face high out-of-pocket costs and as such may be more vulnerable to financial toxicity associated with seeking thyroid care.^15^

### Accounting for Noncompliance and Derivation of Analytic Cohort

Given the known variation in reporting under the Hospital Price Transparency Rule, we created inverse probability weights to adjust for potential selection bias in our data—as has been done in prior work using Turquoise data.^16^ To create these weights, we fit a logistic regression model with the hospital covariates noted above to estimate the probability of a hospital reporting at least one price for a total lobectomy and total thyroidectomy relative to all nonfederal hospitals within the AHA (Table 1). The inverse weights were then calculated and used to give greater weight to prices reported by hospitals that were relatively less likely to report prices for thyroidectomy surgery. All subsequent analyses incorporate these inverse probability weights.

**TABLE 1.** Characteristics of Hospitals Reporting Commercially-Negotiated Prices for Thyroid Lobectomy and Total Thyroidectomy (n = 1299)

Hospital Characteristic	Hospitals Reporting* (n=1,299)	All Hospitals (n = 4280)	*P*
Census region			
Northeast	217 (17%)	536 (13%)	**<0.001**
South	535 (41%)	1709 (40%)	0.407
Midwest	309 (24%)	1178 (28%)	**0.006**
West	238 (18%)	808 (19%)	0.642
System affiliation			
Independent	277 (21%)	1444 (34%)	**<0.001**
In a system	1022 (79%)	2836 (66%)	**<0.001**
No. beds			
0–100	258 (20%)	1418 (33%)	**<0.001**
101–300	352 (27%)	832 (19%)	**<0.001**
>300	689 (53%)	2030 (47%)	**<0.001**
CBSA			
Metropolitan	1027 (79%)	2904 (68%)	**<0.001**
Micropolitan/rural	272 (42%)	2752 (64%)	**<0.001**
Teaching status			
Nonteaching	1184 (91%)	4095 (96%)	**<0.001**
Teaching	115 (9%)	185 (4%)	**<0.001**
Ownership			
For-profit	278 (21%)	1148 (27%)	**<0.001**
Not-for-profit	871 (67%)	2274 (53%)	**<0.001**
Governmental	150 (12%)	858 (20%)	**<0.001**
CoC accreditation			
Not accredited	554 (43%)	921 (22%)	**<0.001**
Accredited	1299 (57%)	3359 (78%)	**<0.001**

Bolded *P* values indicate <0.05 significance.

* Includes hospitals reporting a commercially-negotiated price for thyroid lobectomy (CPT 60620) and total thyroidectomy (CPT 60640).

CBSA, core-based statistical area.

### Thyroidectomy Procedures

CPT codes were used to identify prices for 3 thyroidectomy procedures: 60220 (thyroid lobectomy), 60240 (total thyroidectomy), and 60252 (total thyroidectomy with CND; listed in order of complexity and extent). These CPT codes reflect a wide array of high-volume, elective thyroidectomy procedures and have been utilized in previous investigations of thyroidectomy outcomes.^17,18^ Thyroidectomy procedures were a selected focus given the clear hierarchy and increasing extent of surgery captured by CPT codes. Thyroidectomy procedures were not specified as required by CMS within the 70-service requirement. However, under the expanded service reporting requirement, necessitating an additional 230 procedures, hospitals may choose to report pricing data for high-volume procedures and services, such as thyroidectomies.^13^ Within the price transparency data, we found that a substantial number of hospitals reported commercially-negotiated prices for the thyroidectomy procedures of interest.

### Statistical Analysis

To assess commercially-negotiated prices for thyroidectomies, we performed a cross-sectional analysis with 2 analytical goals. First, descriptive analyses were performed to evaluate the distribution of commercial prices across thyroidectomy surgeries. Because of the known right skew of price data, winsorization was performed at the 1st and 99th percentiles for each thyroidectomy procedure. Additionally, we assessed differences in the prices of thyroidectomy surgeries within the same hospital. To do so, we identified the median commercially-negotiated price for each hospital reporting thyroid lobectomy, total thyroidectomy alone, and total thyroidectomy with CND. We then calculated the ratio of the median price between surgeries to identify differences in the prices of thyroidectomy surgeries (eg, median price of total thyroidectomy:median price of thyroid lobectomy). To avoid potential error in reporting, we only assessed intrahospital comparisons at hospitals that report all 3 thyroidectomy procedures.

Second, we utilized a risk-adjustment model to identify geographic and hospital characteristics that may be associated with commercial price variation. To adjust for potentially appropriate sources of price variation, a multivariable linear regression model was used, which adjusted for thyroidectomy procedure and hospital characteristics, such as census region, health system affiliation, hospital bed size, core-based statistical area, teaching status, hospital ownership, and Commission on Cancer (CoC) accreditation.^19^ Due to prices being reported at the individual hospital level, we clustered standard errors at the hospital level to account for potential intrahospital price correlation.

Sensitivity analyses, which accounted for state variation and Area Wage Index (AWI)-adjustment, were performed given known differences in state policy for commercial insurance price negotiations and adjusted mean differences in negotiated prices observed by census region. An additional sensitivity analysis evaluated market competition using the Health Care Cost Institute’s Herfindahl–Hirschman Index to evaluate for any relationship between a metropolitan area’s market competition and the negotiated prices for thyroidectomy surgery within hospitals (Supplemental Table 1, see http://links.lww.com/AOSO/A485). All analyses were performed using statistical software (Stata 17.0, College Station, TX) at the 5% significance level.

## RESULTS

### Hospital Price Reporting and Patterns

Overall, 1299 hospitals (30.4%) reported a commercial price for thyroid lobectomy and total thyroidectomy alone. These hospitals (n = 1299) were more likely to be located in the Northeast or Midwest, system-affiliated, larger, metropolitan, not-for-profit, and non-CoC accredited compared to all hospitals (n = 4280; Table 1). Of the studied thyroidectomy procedures, the most commonly reported was thyroid lobectomy by 95% (n = 1471/1556) of hospitals reporting a thyroidectomy price (Table 2). About 89% (n = 1378/1556) of hospitals reported prices for total thyroidectomy and 68% (n = 1052/1556) reported prices for total thyroidectomy with CND.

**TABLE 2.** Descriptive Statistics for Commercially-Negotiated Rates in Thyroidectomy (n = 1556 Hospitals)

	Thyroid Lobectomy (CPT 60620)	Total Thyroidectomy (CPT 60640)	Total Thyroidectomy With CND (CPT 60652)
Hospitals reporting, n (%)	1471 (95%)	1378 (89%)	1052 (68%)
Rates, n	27,220	25,723	25,723
wRVU*	11.19	15.04	22.01
Negotiated rates			
Mean	$8844	$9333	$8374
Median (IQR)	$6483 ($2217–$11,443)	$6732 ($2566–$11,321)	$6232 ($3118–$10,916)
Min	$109	$0	$0
Max	$50,645	$59,720	$52,876
Negotiated rates per wRVU			
Mean	$790	$621	$380
Median (IQR)	$579 ($198–$1023)	$448 , ($171–$753)	$283 , ($142–$496)
Min	$10	$0	$0
Max	$4526	$3971	$2402

* Estimated wRVU for thyroidectomy were derived from Ramsey et al^20^ and not Price Transparency data.

IQR, interquartile range; Min, minimum; Max, maximum.

**FIGURE 1.**
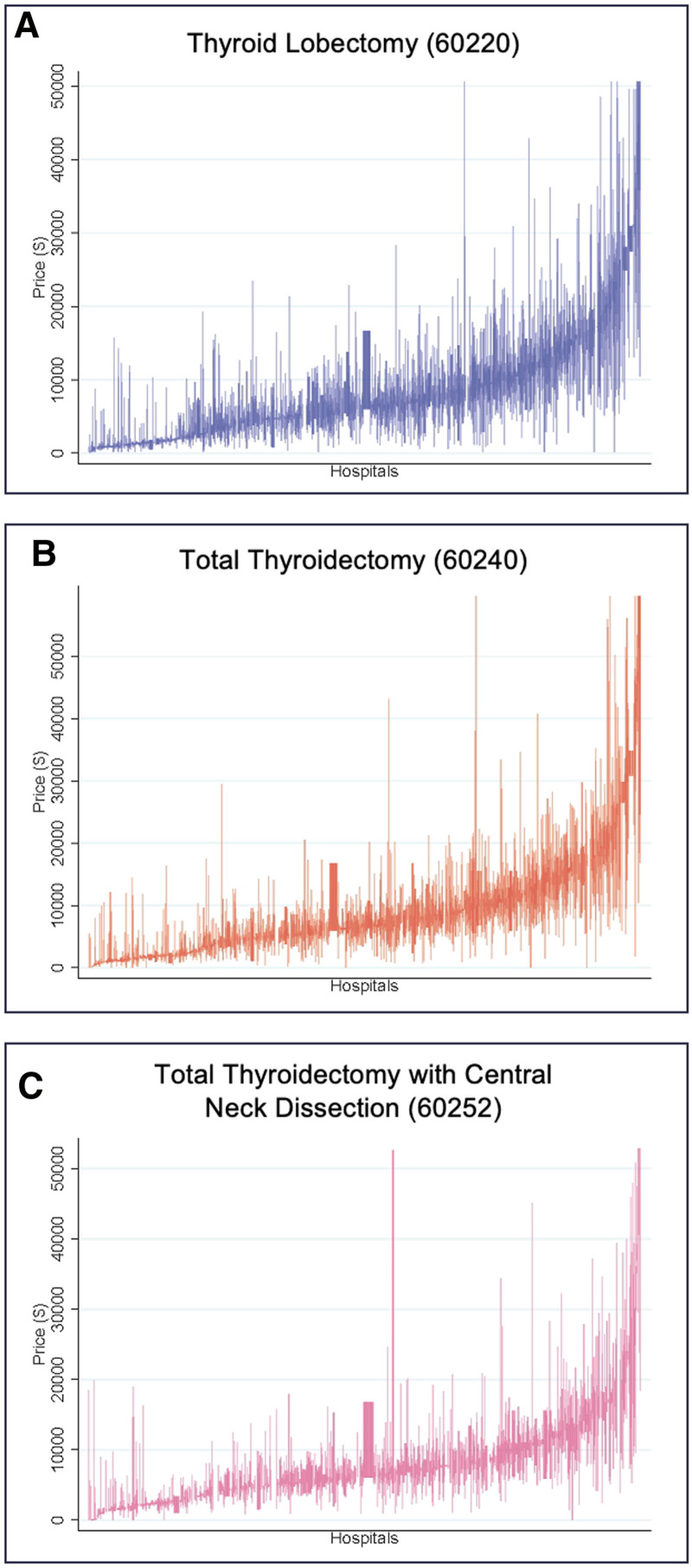
Intrahospital commercially-negotiated prices interquartile ranges by thyroidectomy procedure. The graphs show interquartile ranges of commercially-negotiated prices for thyroidectomy procedures within individual hospitals. There is significant variation within and between hospitals in median prices reported for thyroid lobectomy (A), total thyroidectomy alone (B), and total thyroidectomy with a central neck dissection (C). Rates are 1% winsorized at the procedure level and multiplied by AWI.

Descriptive analysis of negotiated prices revealed median commercially-negotiated prices (interquartile range) were $6483 ($2217–$11,443) for thyroid lobectomy, $6732 ($2566–$11,321) for total thyroidectomy alone, and $6232 ($3118–$10,916) for total thyroidectomy with CND (Table 2). Of hospitals reporting a price for all 3 thyroidectomy procedures (n = 1022), only 32% (n = 323) of hospitals reported median negotiated prices following the hypothesized pattern of increasing price and extent of surgery—from thyroid lobectomy to total thyroidectomy alone to total thyroidectomy with CND. In addition, we found wide intrahospital variation in commercially-negotiated prices within each procedure (Fig. 1).

### Intrahospital Median Commercially-Negotiated Prices

**FIGURE 2.**
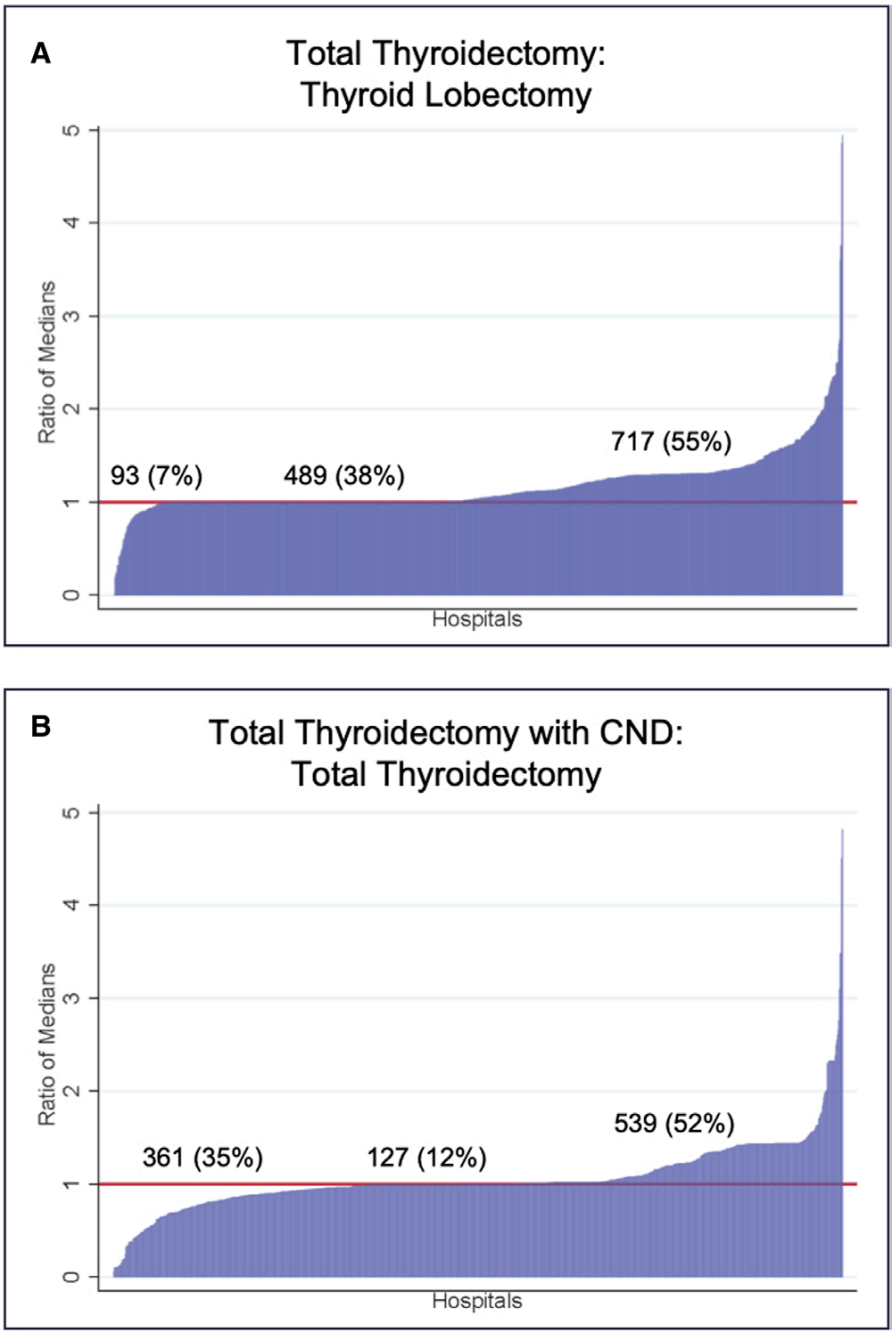
Ratio of intrahospital median commercially-negotiated prices. The graphs depict ratios of intrahospital median negotiated prices for total thyroidectomy compared to thyroid lobectomy (A) and for total thyroidectomy with central neck dissection (CND) compared to total thyroidectomy alone (B). Hospitals below the red line are locations where the median reported negotiated prices are more for less complex surgery (paradoxical pricing). Unexpectedly, at 7% (n = 93) of hospitals total thyroidectomy prices were less than lobectomy, and at 35% (n = 361) total thyroidectomy with CND prices were less than total thyroidectomy alone.

Intrahospital comparison of median commercial prices comparing total thyroidectomy to thyroid lobectomy demonstrated that 7% of hospitals had higher prices for lobectomy compared with total thyroidectomy, while 38% had equal prices and 55% of hospitals had the expected lower prices for lobectomy (Fig. 2). When comparing total thyroidectomy with CND (the most extensive procedure) to total thyroidectomy alone, 35% of hospitals had higher prices for total thyroidectomy alone than with CND, 12% had equal prices, and 53% had the expected higher price for total thyroidectomy with CND (Fig. 2).

### Risk-Adjusted Mean Commercially-Negotiated Prices

Adjusted mean prices for thyroidectomy varied significantly by hospital characteristics (Table 3). Risk-adjusted mean negotiated prices found that not-for-profit hospitals had significantly lower adjusted mean prices compared to for-profit hospitals ($8266 vs $10,625, *P* = 0.022). Procedure type also significantly impacted adjusted mean prices, with total thyroidectomy with CND having lower prices compared to total thyroidectomy alone ($8295 vs $9446, *P* = 0.001). Census region, system affiliation, hospital bed size, teaching status, and CoC accreditation status were not significantly associated with mean commercially-negotiated prices.

**TABLE 3.** Risk-Adjusted Mean Commercially-Negotiated Prices

Hospital Characteristic	Adjusted Mean	95% CI	Adjusted Mean Difference	*P*
Census region				
Northeast	$9657	$7453–$11,861	Ref	Ref
South	$7920	$7040–$8801	$−1737	0.119
Midwest	$7712	$6845–$8579	$−1945	0.12
West	$11,009	$9156–$12,862	$1352	0.345
System affiliation				
Independent	$8113	$6437–$9789	Ref	Ref
In a system	$9267	$8568–$9965	$1153	0.211
No. beds				
0–100	$8729	$7105–$10,353	Ref	Ref
101–300	$8861	$7912–$9809	$132	0.894
>300	$9035	$7882–$10,187	$306	0.782
Core-based statistical area				
Metropolitan	$8855	$7900–$9810	Ref	Ref
Micropolitan/rural	$9004	$7216–$10,792	$149	0.897
Teaching status				
Nonteaching	$8988	$8192–$9784	Ref	Ref
Teaching	$7798	$5889–$9707	$−1190	0.29
Ownership				
For-profit	$10,625	$8937–$12,313	Ref	Ref
Not-for-profit	$8266	$7350–$9183	**$−2359**	**0.022**
Governmental	$8760	$6459–$11,062	$−1865	0.186
Cancer center				
Non-CoC accredited	$8494	$7643–$9345	Ref	Ref
CoC accredited	$10,058	$8508–$11,607	$1564	0.093
Procedure				
Total thyroidectomy	$9346	$8548–$10,144	Ref	Ref
Lobectomy	$8886	$8051–$9721	$−460	0.07
Total thyroidectomy with CND	$8295	$7513–$9077	**$−1052**	**0.001**

Bolded *P* values indicate <0.05 significance.

CI, Confidence interval; CoC, Commission on Cancer; Ref, reference.

**FIGURE 3.**
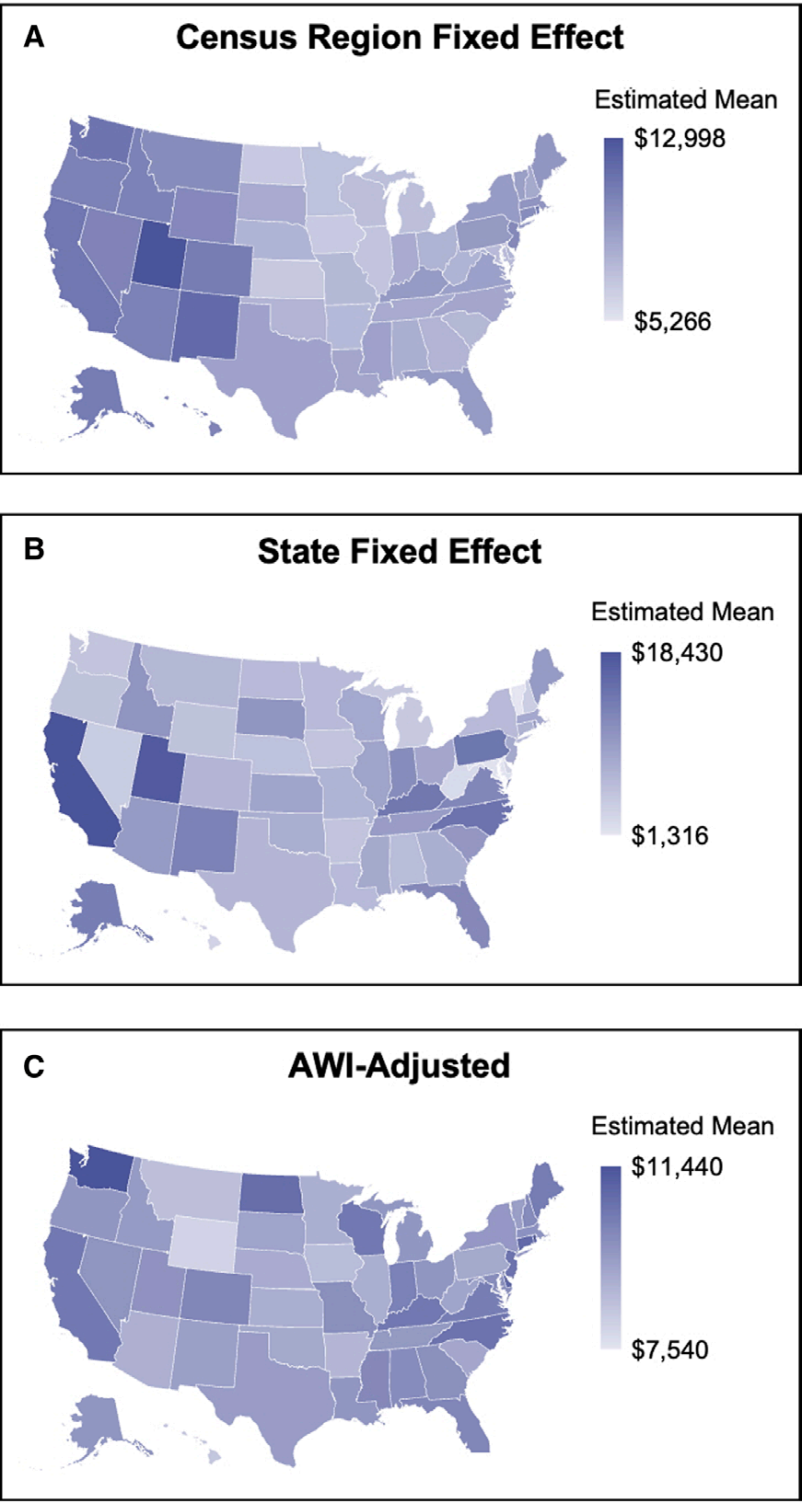
Estimated state mean commercially-negotiated prices for thyroidectomy procedure by census region, state, and AWI-adjustment. The United States (US) maps depict adjusted mean commercially-negotiated prices across thyroidectomy procedures estimated by state with fixed effect at the census region (A), state (B), and AWI-adjustment alone (C).

Given the variation in adjusted mean prices for thyroidectomy at the census region level and known differences in state-level policy, further analyses at the individual state level and with AWI-adjustment alone were performed (Fig. 3). In the state fixed effect model, commercially-negotiated prices across the extent of thyroidectomy ranged from $18,430 in California and $17,551 in Utah to $1316 in Vermont and $2487 in West Virginia.

## DISCUSSION

The present study is the first to our knowledge to comprehensively assess commercially-negotiated price variation across the most commonly performed thyroidectomy procedures and has 3 principal findings. First, there is significant variation in negotiated prices across different thyroidectomy procedures, with hospitals exhibiting higher prices for less extensive surgeries. Second, the price patterns for thyroidectomy procedures do not always follow the expected hierarchy based on the extent of thyroidectomy and procedure complexity. Only 32% of hospitals report prices in the increasing extent of surgery from thyroid lobectomy, then total thyroidectomy alone, followed by thyroidectomy with a CND. Third, hospital characteristics such as geographic location and ownership significantly impact the adjusted mean thyroidectomy prices. Collectively, these findings suggest that reported negotiated prices may not always align with the complexity of the thyroidectomy performed and that negotiated prices vary significantly by hospital factors.

Previous studies have evaluated negotiated price variation for various surgical procedures, but inadequate data exist on thyroidectomy-specific price variation.^21–23^ Prior research on variation in commercially-negotiated thyroidectomy prices was limited by low rates of hospital reporting, inclusion of only National Cancer Institute (NCI)-designated cancer centers, and exclusion for total thyroidectomy with CND.^24–26^ An early study by Xiao et al^24^ presented a cross-sectional analysis of commercial payer-negotiated prices of services for thyroid cancer at NCI-designated cancer centers; however, less than 10 hospitals reported prices for thyroidectomy, limiting analysis and conclusions. In another study evaluating negotiated price variation across oncologic surgeries, including thyroid lobectomy, Rochlin et al^25^ found that lobectomy prices on average varied threefold between insurers at the same hospital. Enumah et al^26^ recently demonstrated that features across hospitals, such as market competition, affect negotiated prices. Our present study expands these findings by incorporating hospital-specific characteristics through the linkage of the Turquoise database with the AHA Annual Survey and demonstrates that significant price variation within individual hospitals exists across 3 of the most common thyroidectomy procedures in a wide range of hospital settings.

The discordance between the extent of thyroidectomy and commercially-negotiated prices for these thyroidectomy procedures is a notable finding. When considering the 3 procedures evaluated, there is a clear hierarchy in the extent of surgery, from thyroid lobectomy to total thyroidectomy alone to total thyroidectomy with a CND, defined by increasing anatomic dissection, surgical complexity, operative time, risk of complications, and resource utilization associated with each procedure.^4,27,28^ This hierarchy is also reflected in the work relative value units (wRVUs) attributed to each thyroidectomy type. The mean wRVU for a thyroid lobectomy is 11.19, for total thyroidectomy 15.04, and for total thyroidectomy with CND 22.01.^20^ However, our analysis reveals paradoxical pricing at 68% of hospitals where negotiated prices for thyroidectomy do not align with this expected pattern. This discrepancy is further elucidated in our risk-adjusted model, which shows total thyroidectomy with CND is $1052 less than total thyroidectomy alone and $591 less than thyroid lobectomy despite it being a more complex and invasive operation.

The discrepancies in negotiated prices and surgical complexity, or paradoxical pricing, may be the result of bundled services that obscure the direct relationship between procedural complexity and reimbursement rates. It is also possible that the discrepancy is related to payer–hospital contract negotiations and because total thyroidectomy with CND is less common than total thyroidectomy, it may be less frequently included in negotiations. Particularly in thyroid cancer, it is possible that inverse pricing for complexity in thyroid surgery could entire surgeons to perform simpler operations and neglect neck dissections when indicated. Overall, these findings underscore the need for greater transparency and standardization in the price negotiation process to ensure that prices more accurately reflect the underlying procedure complexity and resource utilization of surgical procedures. Surgeon involvement in negotiations and advocacy for greater granularity in price reporting pose additional avenues to address this issue. Policymakers can use these findings to regulate insurer negotiations and prevent cost discrepancies that contribute to financial hardship for patients undergoing thyroidectomy. Further research is also needed to determine if the paradoxical pricing phenomenon is present in other disease processes or solid organ cancers with increasing surgical complexity CPT codes.

This study also found variations in negotiated thyroidectomy prices based on hospital geographic location and ownership. While some variation in negotiated price may be expected along geographic lines, even after area wage adjustment, we found significant, unexpected variation in negotiated rates for thyroidectomy. Our finding is that hospital location by state has unexplained differences in negotiated prices. For example, those hospitals located in the Western region of the United States had higher negotiated prices by $1352 across all thyroidectomy procedures. This finding is consistent with prior work evaluating actual costs related to thyroidectomy. Reinke et al^29^ showed there to be a 27% variation in total thyroidectomy costs across US states not explained by patient, facility, or state-level factors. Furthermore, in a study looking at factors associated with actual hospital costs postthyroidectomy, hospital location in the Western region was associated with increased hospital costs compared with other US regions.^30^ Together with our findings, these data suggest that other elements such as insurance competition, market dynamics, and resource utilization may be driving these regional price differences. Further research is needed to identify the specific factors contributing to these regional disparities and to develop strategies for promoting more equitable pricing across different geographic areas.

### Limitations

The study should be interpreted in the context of several limitations. First, the analysis relies on self-reported price data from hospitals, which may be subject to errors. Inherent in the data are the inconsistencies in the interoperability and reporting of machine-readable price transparency files. Additionally, hospitals may report prices for thyroidectomy procedures not offered at their facility. However, the use of a large, national database helps mitigate the impact of potential reporting errors. Second, the study does not account for patient-level factors, such as insurance plan details or out-of-pocket costs, which may influence the actual prices paid by patients. Furthermore, this study is unable to assess surgeon-level factors, such as surgeon training, which may explain some price variation due to differing funds-flow rates by specialty. Nevertheless, the findings provide valuable insights into the variation of negotiated prices at the hospital level and the discordance of the extent of thyroidectomy with commercially-negotiated prices. Third, the cross-sectional nature of the study precludes the assessment of longitudinal trends in price variation. Future research should explore the impact of price transparency on negotiated rates over time, patient choice of center, and rates of financial toxicity. Finally, there may be additional unmeasured factors, such as hospital negotiation power, which may explain the price variation for which we observed. While additional granular data pertaining to the negotiating power of hospitals and insurers is limited, we risk-adjusted for a variety of hospital-level factors that have been shown to influence the price of surgery.

## CONCLUSIONS

Our study reveals that the complexity of thyroid surgical procedures is not reflected in the price-negotiated rates paid by insurers to hospitals. For most hospitals, they are paid less when taking on more complex procedures. These findings underscore concerns about fair reimbursement to hospitals and the potential of the Price Transparency Rule to illuminate unwarranted differences in negotiated rates. From a patient perspective, these findings highlight the challenges in making meaningful, informed decisions based on price alone especially if they are underinsured. Policymakers and payers should consider these findings when developing strategies to promote more consistent and equitable pricing for surgical procedures. Efforts to improve price transparency and align negotiated prices with procedure complexity will be critical in addressing the financial burden faced by patients with thyroid conditions and ensuring access to high-quality, affordable care.

## Supplementary Material


