# Supplementary material for: Sometimes Less Is More: A Cross-Sectional Analysis of Commercially-Negotiated Price Variation for Thyroidectomy
Source: Ann Surg Open. 2025 Mar 12;6(1):e564. doi: 10.1097/AS9.0000000000000564 (PMC11932599; doi:10.1097/AS9.0000000000000564)
Supplement: Supplementary file 1 [file as9-6-e564-s001.pdf]

Supplemental Digital Content, Table 1: Intrahospital Median Comparison for Thyroidectomy Procedure by Herfindahl-Hirschman Index

	**Unconcentrated** (<1500)	**Moderately Concentrated** (1500-2500)	**Highly Concentrated** (2500-5000)	**Very Highly Concentrated** (>5000)
**Total Thyroidectomy (60240) vs. Thyroid Lobectomy (60220)**				
**60240 < 60220**	14 (5%)	13 (6%)	28 (9%)	3 (13%)
**60240 = 60220**	108 (38%)	75 (37%)	114 (36%)	6 (25%)
**60240 > 60220**	162 (57%)	116 (57%)	176 (55%)	15 (63%)
**Total Thyroidectomy with Central Neck Dissection (60252) vs. Total Thyroidectomy (60240)**				
**60252 < 60240**	94 (41%)	48 (29%)	109 (42%)	9 (45%)
**60252 = 60240**	41 (18%)	16 (10%)	36 (14%)	0 (0%)
**60252 > 60240**	95 (41%)	99 (61%)	115 (44%)	11 (55%)
